# APOC1 is a prognostic biomarker associated with M2 macrophages in ovarian cancer

**DOI:** 10.1186/s12885-024-12105-z

**Published:** 2024-03-21

**Authors:** Shimin Yang, Jingxiao Du, Wei Wang, Dongmei Zhou, Xiaowei Xi

**Affiliations:** 1 Division of Gynecologic Oncology, Department of Gynecology and Obstetrics, Shanghai General Hospital, Shanghai Jiao Tong University School of Medicine, No. 650 Xin Songjiang Road, Fang Song Street, Songjiang District, City Shanghai, China; 2 Department of Ophthalmology, Shanghai General Hospital, Shanghai Jiao Tong University School of Medicine, City Shanghai, China

**Keywords:** APOC1, Ovarian cancer, Immune response, M2 TAMs, Biomarker

## Abstract

**Background:**

Recent studies have demonstrated that APOC1 is associated with cancer progression, exerting cancer-promoting and immune infiltration-promoting effects. Nevertheless, there is currently no report on the presence of APOC1 in ovarian cancer (OV).

**Method:**

In this study, we conducted data analysis using the GEO and TCGA databases. We conducted a thorough bioinformatics analysis to investigate the function of APOC1 in OV, utilizing various platforms including cBioPortal, STRING, GeneMANIA, LinkedOmics, GSCALite, TIMER, and CellMarker. Additionally, we performed immunohistochemical staining on tissue microarrays and conducted in vitro cellular assays to validate our findings.

**Result:**

Our findings reveal that APOC1 expression is significantly upregulated in OV compared to normal tissues. Importantly, patients with high APOC1 levels show a significantly poorer prognosis. Furthermore, our study demonstrated that APOC1 exerted a crucial function in promoting the capacity of ovarian cancer cells to proliferate, migrate, and invade. Additionally, we have identified that genes co-expressed with APOC1 are primarily associated with adaptive immune responses. Notably, the levels of APOC1 in OV exhibit a correlation with the presence of M2 Tumor-associated Macrophages (TAMs).

**Conclusion:**

APOC1 emerges as a promising prognostic biomarker for OV and exhibits a significant association with M2 TAMs in OV.

**Supplementary Information:**

The online version contains supplementary material available at 10.1186/s12885-024-12105-z.

## Introduction

Ovarian cancer (OV) is the second most common malignant tumor and has the highest mortality rate among reproductive system tumors in women [1]. The most prevalent subtype of ovarian cancer is High-Grade Serous Ovarian Cancer (HGSOC), with the majority of cases originating from the fallopian tube epithelium. HGSOC has a low 5-year survival rate of only 30% primarily because most patients are diagnosed at stage III (51%) or stage IV (29%) [2, 3]. As a result, the therapy for HGSOC has changed little over the decades and remains surgical treatment combined with radiation and chemotherapy. Patients with advanced ovarian cancer and mutations in the tumor suppressor genes BRCA1/2 are often treated with maintenance therapy involving Poly (ADP-ribose) polymerase (PARP) inhibitors, such as Olaparib [4, 5]. Nevertheless, not all patients will respond favorably to immunotherapy, and those with OV have limited responses. Therefore, it is crucial to deeply understand the mechanisms of ovarian cancer development and progression to develop improved early diagnostic and therapeutic strategies. Currently, immunotherapy for OV mainly utilizes programmed death-ligand 1 (PD-L1) immune checkpoint inhibitor, but the effect is not obvious. The reason for this lack of clarity is that the targets and mechanisms of effect of OV drugs are poorly understood. Recent research has indicated that the effectiveness of immunotherapy in OV could be linked to the abundance of CD8 + T cells [6, 7]. Even based on the level of T cells, OV has been categorized into three distinct types: T-cell inflamed or “hot” tumors, characterized by infiltration of T cells within deposits (islets) of malignant cells and peripheral stroma; excluded tumors, where T cells are confined to the stroma and absence in the deposits of malignant cells; and non-inflammatory or “immune deserts” or “cold” tumors. These different types exhibit varying degrees of responsiveness to immune checkpoint blockade. Among ovarian cancer subtypes, HGSOC exhibits the highest infiltration of CD8 + T cells, which is associated with a more favorable prognosis [8]. However, the current clinical application of immunotherapy does not take into consideration the immunophenotype. During a phase III clinical trial, the first-line treatment involving the combination of the anti-PDL1 antibody atezolizumab, carboplatin, paclitaxel, and bevacizumab revealed that only approximately 20% of patients who derived benefits exhibited high PD-L1 positivity [9]. Nevertheless, the immune environment is a complex and synergistic system that cannot be accurately explained by a single factor. For instance, ovarian cancer recruits immature myeloid cells and M2 macrophages, which downregulate the expression of CD80, CD86, and IL-12 in effector T cells. Consequently, the identification of additional biomarkers will aid in the selection of appropriate candidates for immunotherapy and facilitate the investigation of mechanisms underlying tumor immunity.

Apolipoprotein C (APOC) is the most abundant apolipoprotein in very low-density lipoprotein (VLDL) cholesterol and is also present in small amounts in low-density lipoprotein (LDL) and high-density lipoprotein (HDL) cholesterol. In humans, there are four different isoforms. Apolipoprotein C1 (APOC1) is a single-chain protein consisting of 83 amino acids initially synthesized in the endoplasmic reticulum. Its molecular weight is 9.3 kDa. The peptide chain undergoes shearing to produce mature APOC1, which contains 57 amino acids and has a molecular weight of 6.6 kDa, making it the smallest apolipoprotein [10]. APOC1 is engaged in normal biological processes and disease development. It is found in chylomicrons (CM), VLDL, and HDL, and serves as an exchange apolipoprotein between various lipoproteins, playing a vital role in lipid homeostasis [11]. Additionally, APOC1 plays an essential role in diabetes, atherosclerosis, and Alzheimer’s disease [12, 13]. Recently, studies on APOC1 have increasingly emphasized its involvement in cancer. In the study of breast cancer, APOC1 has been identified as a potential diagnostic and classification biomarker [14]. Furthermore, APOC1 has been found to promote the development of gastric cancer [15]. In a study on glioblastoma, APOC1 was discovered to enhance tumorigenesis by reducing KEAP1/NRF2 and CBS-regulated ferroptosis [16]. Recently, it has been revealed that APOC1 can be used as an immunological biomarker, influencing macrophage polarization and facilitating renal cell cancer [17]. In hepatocellular carcinoma, a single-cell RNA sequencing study demonstrated that inhibiting APOC1 promoted the transformation of M2 macrophages into M1 macrophages via the ferroptosis pathway, thereby enhancing anti-PD1 immunotherapy [18]. However, the function of APOC1 in OV hasn’t been reported.

The aim of this research was to survey the function of APOC1 in OV as well as to evaluate its potential as an immune biomarker.

## Materials and methods

### Data resources

The fragments per kilobases per million mapped fragments (FPKM) files containing clinical information were derived from The Cancer Genome Atlas (TCGA). Expression matrix data in SOFT format, including GSE12470 [19], GSE66957 (Cheng et al., unpublished data, 2015), GSE10971 [20] and GSE52037 [21], were obtained from the Gene Expression Omnibus (https://www.ncbi.nlm.nih.gov/geo/) (GEO). Somatic mutation and copy number variation (CNV) analyses of APOC1 in OV were carried out using the cBioPortal website (www.cbioportal.org) [22] (Date of data acquisition and analysis: 2023-06-11). Additionally, Kaplan-Meier overall survival analysis of APOC1 expression in ovarian cancer patients was performed using the Kaplan-Meier Plotter tool (https://kmplot.com/analysis/) [23, 24] (Date of data acquisition and analysis: 2023-06-11).

### Analysis of differentially expressed genes

The differential analysis of genes was conducted using the limma R package (version 3.52.4) [25]. DEGs were identified for genes with *p* < 0.05 &|log2FC|≥1.

### Protein-protein interaction analysis

The protein-protein interaction (PPI) network analysis of APOC1 was performed using the online tool STRING 11.0 b (https://string-db.org/) [26] (Date of data and analysis and analysis: 2023-06-11). In order to further investigate these relationships between interacting genes further, we utilized the online tool GeneMANIA (http://www.genemania.org) [27] (Date of data acquisition and analysis: 2023-06-13).

### LinkedOmics and GSCALite

The genes significantly associated with APOC1 were identified through screening TCGA data, and these genes were enriched using the online tool LinkedOmics (www.linkedomics.org) [28] (Date of data acquisition and analysis: 2023-06-13). Subsequently, pathway activity analysis was conducted using the online tool GSCALite (www.bioinfo.life.hust.edu.cn/web/GSCALite/) [29] (Date of data acquisition and analysis: 2023-06-13).

### Immune infiltration analysis

The immune correlation assessment was performed using the ssGSEA algorithm provided in the GSVA R package (version 1.44.5) [30]. In order to calculate the immune infiltration corresponding to the TCGA data, markers for 24 immune cells were utilized [31]. The analysis of the correlation between APOC1 expression and immunity cells was performed utilizing the TIMER 2.0 platform (http://timer.cistrome.org/) [32] (Date of data acquisition and analysis: 2023-08-18). Specifically, CD206, CD163, IL10, and ARG1 were selected as markers for macrophage M2.

### Single-cell RNAseq data analysis

The single-cell sequencing data (GSE147082 [33] and GSE165404 [34]) were obtained from the GEO. Cell marker analysis of single cell sequencing data utilizing the online tool CellMarker 2.0 (http://biocc.hrbmu.edu.cn/CellMarker/) [35] (Date of data acquisition and analysis: 2023-08-25).

### Clinical sample

Between 2017 and 2022, a total of 61 HGSOC tissues and 13 fallopian tubes (FT) tissues were obtained from Shanghai General Hospital. Prior to the operations, patients were informed about the procedures and provided their consent. Ethical approval for the study was obtained from the Medical Ethics Committee of Shanghai First People’s Hospital. Informed consent was taken from participants to participate in the study.

### Production of issue microarray

The tissue samples were fixed in tissue fixative for more than 24 h. Subsequently, the tissues were trimmed using a scalpel in a fume hood and placed in embedding cassettes with appropriate labels. To remove water from the tissues, they were sequentially immersed in different concentrations of ethanol and xylene for dehydration. The dehydrated tissues were then immersed in preheated liquefied wax and left to soak overnight. Using the HistoCore Arcadia system (HistoCore Arcadia, Leica, Germany), tissue wax blocks were created. Finally, the prepared tissue wax blocks were sent to Servicebio (Wuhan, China) for tissue microarray production. The tissue samples were initially stained with HE and then localized by two pathologists before being sampled for tissue microarrays. Subsequently, the scoring of the IHC staining was performed by ImageJ software, as described in the Immunohistochemistry section in methods.

### Immunohistochemistry (IHC)

To melt the surface sealing wax, the tissue microarray was placed in a Thermostatic Incubator (DNP-9052, JINGHONG, Shanghai) at 60 °C overnight. Deparaffinization and rehydration of the tissue microarrays were carried out by immersing them in xylene and ethanol concentration gradients in a fume hood. For antigen retrieval Citrate Antigen Extraction Solution (P0081; Beyotime, China) was used in boiling water for 7 min. To eliminate endogenous peroxidase activity, a 3% hydrogen peroxide solution was applied, and the tissues were then sealed with 10% goat serum (C0265; Beyotime, China) for 1 h. After rinsing with PBS, the tissue microarrays were incubated with the anti-APOC1 antibody (EPR16813; Abcam, UK) diluted to 1:800, the anti-CD163 antibody (16646-1-AP, Proteintech, China) diluted to 1:1000 and the anti-CD206 antibody (60143-1-Ig, Proteintech, China) diluted to 1:10000 at 4 °C overnight. The Immunohistochemistry Kit (GK500705; Genentech, China) was used for secondary antibody incubation and staining of the tissue microarrays. Hematoxylin (C0107; Beyotime, China) was applied for staining the tissue microarrays for 3 min, followed by termination in distilled water. Sequential dehydration of the tissue microarrays was achieved by immersing them in a gradient solution of xylene and ethanol. Finally, the slices were closed with neutral resin (GT100519; GeneTech, China). The figures were captured utilizing a microscope (Leica, London, UK). The obtained results were analyzed using ImageJ software (version 1.52a; National Institutes of Health) [36] and the IHC Profiler plugin. Cytoplasmic staining scores were calculated using Plugins -> IHC Profiler->Cytoplasmic Stained Image->H DAB, while nuclear staining scores were obtained by Plugins -> IHC Profiler->Nuclear Stained Image->H DAB->Set Threshold, followed by Plugins -> Macros -> IHC Profiler. Finally, Immunohistochemical score = cytoplasmic staining score * nuclear staining score. The four IHC Profiler scores were defined as follows: 4: High positive (gray scale: 0–60); 3: Positive (gray scale value: 61–120); 2: Low Positive (gray scale value: 121–180); 1: Negative (gray scale value: 181–236).

Given that our tissue microarrays primarily target regions with a high concentration of tumor cells, which could potentially influence the IHC results, we conducted an analysis of the correlation between APOC1 expression and the expression of CD163 and CD206 using the average optical density (AOD) values obtained through Image-Pro Plus (version 6.0; Media Cybernetics, Rockville, MD). The procedure is as follows:

1. Opening the picture, click measure -> intensity, click new -> std. optical density -> options -> image in the intensity box, and then selecting the blank place in the figure->ok.
2. Changing the incidental level to the value of blank place and then clicking measure->count/size->selecte colours->click the icon of the pen.
3. Selecting the immunohistochemistry picture of the positive protein expression region, and clicking close after the selection.
4. Finally getting the mean of area and mean of Integrated Optical Density (IOD) by clicking Measure->Select Measurements, selecting iod->ok->count inside and clicking view->statistic.

AOD = mean of IOD/ mean of area.

### Cell growth and cell culture

Hey, Caov3, 293T, and tubal epithelial cells OE E6/E7 were acquired from the National Collection of Authenticated Cell Cultures (Shanghai, China). THP-1 cells were provided by Servicebio (Wuhan, China). Hey, Caov3, 293T, and OE E6/E7 were cultured in high-sugar DMEM medium (319-005-CL; MUTICELL, China) with 10% fetal bovine serum (086–150; MUTICELL, China). THP-1 cells were cultured in 1640 DMEM medium (350-000-CL; MUTICELL, China) with 10% fetal bovine serum (086–150; MUTICELL, China). The cells were cultured in a growth environment of 37 °C and 5% CO2 in a CO2-Incubator (51,023,126, Thermo Fisher Scientific, USA).

### Plasmid construction and transfection

The plasmids designed for APOC1 knockdown were synthesized by Genomeditech (Shanghai, China) and were validated through DNA sequencing. To generate the shAPOC1 plasmid, the shRNA primer pair specific for APOC1 was derived from the shRNAlibrary (TRC) and inserted into the pLKO.1-Puro vector. Cells transfected with the APOC1 knockdown plasmids were labeled as shAPOC1-1 and shAPOC1-2, while cells transfected with the control plasmid were labeled as shNC. 293T cells were cultured in 6 cm dishes until reaching a cell density of 50%. Transfection was carried out using Lipofectamine 3000 reagent (Invitrogen Life Technologies, USA) based on the protocol provided by the manufacturer. The 293T cells were then incubated at 37 °C with 5% CO2 for 48 h. The supervisory fluid was gathered and centrifuged to obtain the lentiviral solution. This lentiviral solution was added to Hey and Caov3 cells in six-well plates at a 40% cell density with varying concentration gradients. The cells were subsequently incubated at 37 °C with 5% CO2 for 48 h, followed by replacement with 4 ml of DMEM containing puromycin (2 mg/ml) for screening. After 24–48 h, the cells with the highest viability in the six-well plate were selected for expansion. Subsequent validation was conducted using the Western Blot assay. The sequence of the short hairpin ribonucleic acid (shRNA) used is as follows:

shAPOC1-1: 5′- GACATTTCAGAAAGTGAAGGA − 3′.

shAPOC1-2: 5′- GCTGAAGGAGTTTGGAAACAC − 3′.

### Western blotting

Protein extraction was performed using RIPA lysis buffer (P0013C; Beyotime, China) supplemented with 1% phenylmethanesulfonyl fluoride (ST507-10 ml; Beyotime, China). The protein lysates were separated with a 15% PAGE Gel Fast Preparation Kit (Epizyme, China) and subsequently transferred to methanol-activated polyvinylidene fluoride membranes (PVDF; Millipore, USA). After membrane transfer, the membranes were placed in 5% skimmed milk (E504BA0014, BBI Life Sciences, China) at room temperature for 1 h. Subsequently, the membranes were rinsed three times with 0.1% Tris-HCl plus Tween-20 (TBST) for 5 min each. After specific detection of the target antigen confirmation, we cut between 25 kDa − 35 kDa and incubated the upper half with anti-GAPDH polyclonal antibody and the lower half with anti-APOC1 polyclonal antibody. In the article show images we marked with red boxes. Images of specific detection of the target antigen and original blots can be seen in the Supplementary file. The membranes were then placed with primary and corresponding secondary antibodies, followed by three additional rinses. Detection was carried out using the ECL luminescence kit (BioVision, USA) on a chemiluminescence imaging system (Tanon 5200; Tanon, China). The antibodies and their respective dilutions used in the experiments were as follows:

Anti-APOC1 antibody (ab205718; Abcam, UK) diluted to 1:1000.

Anti-GAPDH antibody (60004-1-lg; Proteintech, China) diluted to 1:50000.

Anti-rabbit (SA00001-2; Proteintech, China) diluted to 1:2000.

Anti-mouse (SA00001-1; Proteintech, China) diluted to 1:2000.

Quantitative comparison of the relative protein levels was performed using ImageJ software (Version 1.52a; National Institutes of Health).

### RNA extraction and qPCR

Total RNA extraction from THP-1 cells was performed using TRIeasy™ LS Total RNA Extraction Reagent (19201ES60; Yeasen, China) following the manufacturer’s protocol. Subsequently, cDNA synthesis was carried out using a reverse transcription reagent (R202-02; EnzyArtisan, China). The mRNA expression levels were normalized to GAPDH and calculated using the 2-ΔΔCt method [37]. The primer sequences for qPCR and the cycling conditions can be found in Table S1.

### Cell proliferation assay

The sh-APOC1 cells were seeded into 96-well plates with a density of 2000 cells per well and cultured for 0, 24, 48, and 72 h. Cell proliferation capacity was evaluated with the Cell Counting Kit-8 (C0038; Beyotime, China) according to the manufacturer’s instructions. After 1 h of incubation, the optical density (OD) values were measured at 450 nm using a multifunctional enzyme marker (VLBLATGD2; Thermo Fisher Scientific, USA). A blank control consisting of DMEM with 10% FBS was used.

### Colony-formation assay

The sh-APOC1 cells were cultured in six-well plates with a seeding density of 800 cells/well for Hey cells and 1500 cells/well for Caov3 cells. The plates were placed in a 37 °C incubator with 5% CO2 for 14 days. After the cultured period, the cells were fixed with 4% paraformaldehyde (BL539A; Biosharp, China) for 30 min. Subsequently, the cells were stained with crystal violet staining solution (C0121; Beyotime, China) for 20 min.

### Transwell cell migration and invasion assays

For the Transwell cell migration assay, 200 µl of serum-free medium containing 1 × 10^5^ Hey cells or 200 µl of serum-free medium containing 1 × 10^5^ Caov3 cells were added to the upper chamber (14,421,030; Corning Incorporated, USA). 700 µl of DMEM medium with 10% FBS was placed in the lower chamber and cultured for 24 h. (Caov3 cells: 48 h). After the incubation period, the cells were fixed with 4% paraformaldehyde for 30 min and subsequently stained with crystal violet staining solution for 20 min.

For the Transwell cell invasion assay, 60 µl of Matrigel substrate (356,234; Corning Incorporated, USA), diluted 1:6 in serum-free medium, was added to the upper chamber of the transwell and allowed to solidify for 1 h at 37 °C before use. Cells and medium were added to the transwell chamber as in the cell migration assay. Finally, after culturing for 48 h (Caov3 cells: 72 h), the cells were fixed and stained.

### Generation and differentiation of macrophages

THP-1 cells were pre-treated with 100 ng/ml PMA (S1819-1 mg; Beyotime, China) for 24 h to induce the generation of M0 macrophages. These M0 macrophages were subsequently stimulated with 20 ng/ml IL-13 (P5178-10 µg; Beyotime, China) and 20 ng/ml IL-4 (P5129-5 µg; Beyotime, China) for 48 h to differentiate into M2 macrophages. The M0 macrophages were then co-cultured with HGSOC cells (Hey or Caov3) in a 6-well transwell chamber with a pore size of 0.4 μm (3470; Corning Incorporated, USA). After co-culturing for 48 h, the macrophages were collected to obtain Tumor-associated Macrophages (TAMs) for qPCR analysis.

### Flow cytometry

Induced macrophages from 6-well plates were collected, centrifuged, washed once with PBS, and then centrifuged again to obtain a single-cell suspension. The cells were subsequently treated with Permeabilization Buffer (00-8333-56, Thermo Fisher Scientific, USA) following the manufacturer’s protocol. Following this, they were incubated with FITC Mouse Anti-human CD163 (333,617, BioLegend, USA), APC Mouse anti-Human CD206 (321,109, BioLegend, USA), and PE Mouse anti-Human ARG1 (369,703, BioLegend, USA) at 4 °C for 30 min. Finally, the stained cells were washed once, resuspended in 200 µl PBS solution, and analyzed on a Fortessa flow cytometer (BD, Biosciences, USA) using FlowJo software (version 10.0, FlowJo, USA).

### Statistical analysis

All experiments in this study were repeated three times. All of our data were initially assessed for normal distribution using the Shapiro-Wilk test. For normally distributed data, unpaired and two-tailed student’s t-tests were conducted. For non-normally distributed data, the Mann-Whitney test was used. All statistical analyses were performed using SPSS software (version 19.0; Chicago, IL, USA). R (version 4.0.3; https://www.r-project.org/) was utilized to analyze data from TCGA and GEO. All graphs were plotted using GraphPad Prism software (version 8.0; San Diego, CA). All experiments were repeated three times. *p* < 0.05 was deemed statistically significant (*p < 0.05, **p < 0.01, ***p < 0.001).

## Results

### The expression of APOC1 was upregulated in human OV compared to FT

**Fig. 1:**
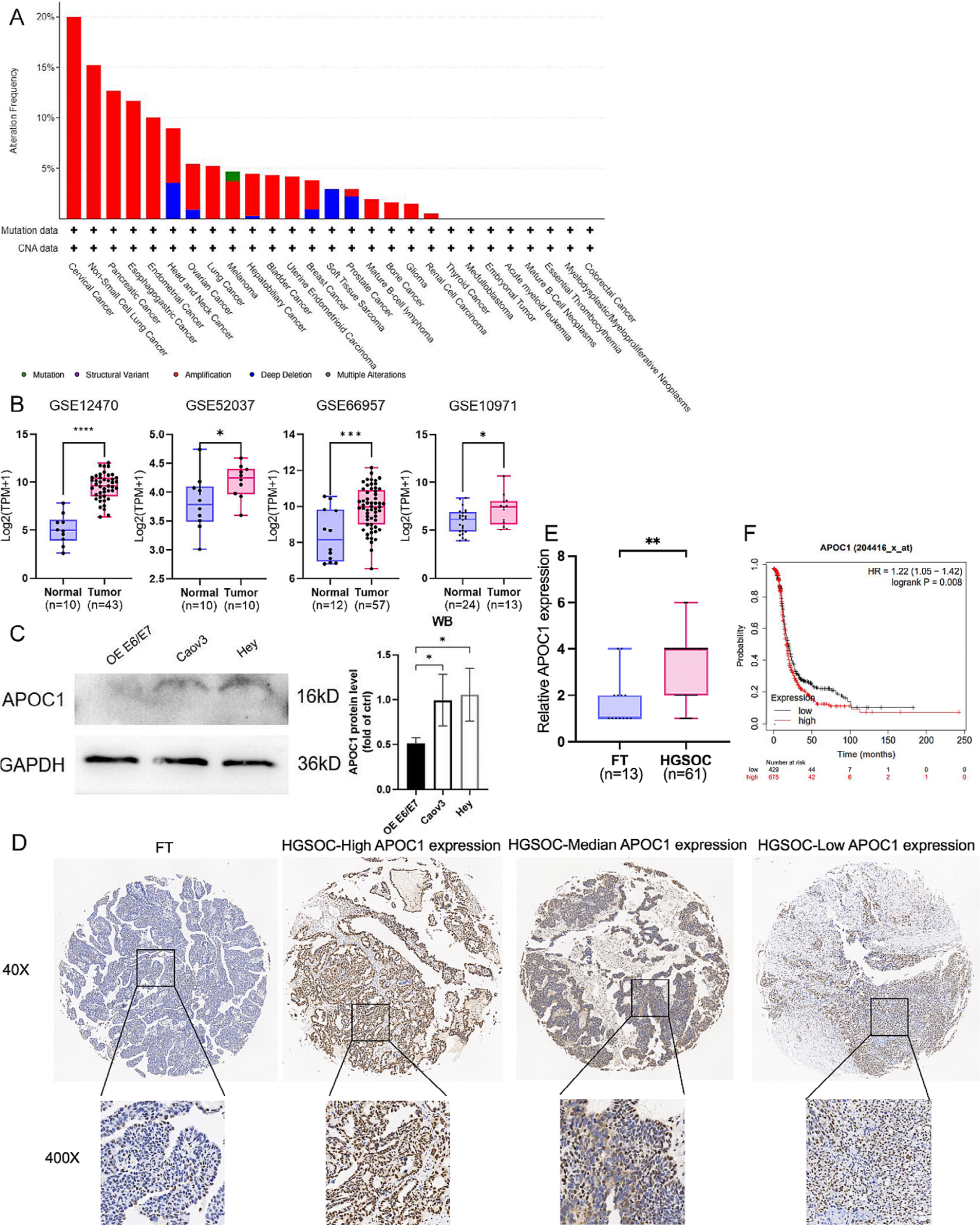
The expression of APOC1 in HGSOC. (**A**) Mutation, structural variant and CNV of APOC1 in pan-cancer. The figure was downloaded from the cBioPortal website (www.cbioportal.org). (**B**) Differential expression analysis of APOC1 in GEO dataset GSE12470, GSE66957, GSE10971 and GSE52037. (**C**) Western Blot assay of APOC1 in Hey, Caov3 and OE E6/E7 cells. (Uncropped images can be found in the Supplementary file) (**D**) Representative figures of IHC staining of APOC1 expression levels in tissue microarray (×40: scale bar = 100 μm; ×400: scale bar = 10 μm). (**E**) Differential expression analysis of APOC1 between HGSOC and FT. (**F**) Kaplan–Meier OS analysis of APOC1 expression in OV patients from Kaplan-Meier Plotter (https://kmplot.com/analysis/). *: *p* < 0.05. **: *p* < 0.01 ***: *p* < 0.001

First, the mutation and amplification of APOC1 rank as the sixth highest in OV compared to pan-cancer (Fig. 1A). By analyzing the GEO datasets GSE12470, GSE66957, GSE10971, and GSE52037, we observed elevated expression of APOC1 in OV (Fig. 1B). Furthermore, through Western Blot assay, APOC1 expression was found to be elevated in the OV cell lines Hey and Caov3 compared to the fallopian tube epithelial cell line OE E6/E7 (Fig. 1C). Consequently, the Hey and Caov3 cell lines were selected for vitro assays. Additionally, immunohistochemical staining of the HGSOC tissue microarray demonstrated increased APOC1 expression in HGSOC compared to FT (Fig. 1D-E, S1A). According to the immunohistochemical scores acquired by IHC, we classified these patients in two groups: the APOC1 hyper-expression group (*n* = 35) and the lower-expression group *n* = 26). Furthermore, there was no significant correlation between APOC1 expression and age, FIGO stage, CA125, HE4, lymph node metastasis, large omental metastasis, ascites, BRCA1/2 mutation and residual tumor size (*P* > 0.05) (Table 1). The Kaplan-Meier overall survival analysis revealed that ovarian cancer patients with high expression of APOC1 exhibited poorer overall survival compared to patients with low expression of APOC1 (Fig. 1F). In conclusion, these findings indicate that APOC1 is high-expressed in OV and is related to poor patient prognosis.

**Table 1:** Basic features of patients in HGSOC tissues and the association between APOC1 expression and clinicopathologic features

Characteristics		Total	Expression of APOC1		P-value
			High (35)	Low (26)	
Age					
< 50		7	5 (71.4%)	2 (28.6%)	0.42444
≥ 50		54	30 (55.6%)	24 (44.4%)	
FIGO staging					
I-II		8	5 (62.5%)	3 (37.5%)	0.75303
III-IV		53	30 (56.6%)	23 (43.4%)	
CA125					
< 30.2		2	2 (100%)	0 (0%)	0.21521
≥ 30.2		59	33(55.9%)	26(44.1%)	
HE4					
< 140		13	7 (53.8%)	6 (46.2%)	0.77195
≥ 140		48	28 (58.3%)	20 (41.7%)	
Lymph node metastasis					
Positive		32	19 (59.4%)	13 (40.6%)	0.74014
Negative		29	16 (55.2%)	13 (44.8%)	
Greater omentum metastasis					
Positive		45	26 (57.8%)	19 (42.2%)	0.91647
Negative		16	9 (56.3%)	7 (43.7%)	
Ascites					
Positive		53	32 (60.4%)	21 (39.6%)	0.22253
Negative		8	3 (37.5%)	5 (62.5%)	
BRCA1/2 mutation					
Positive		16	8 (50%)	8 (50%)	0.48707
Negative		45	27 (60%)	18 (40%)	
Residual tumor size					
R0		25	12 (48%)	13 (52%)	0.21717
>R0		36	23 (63.9%)	13 (36.1%)	

Immunohistochemical score > 3 is considered high expression

### APOC1 enhances the proliferation, migration and invasion of OV cells in vitro

**Fig. 2:**
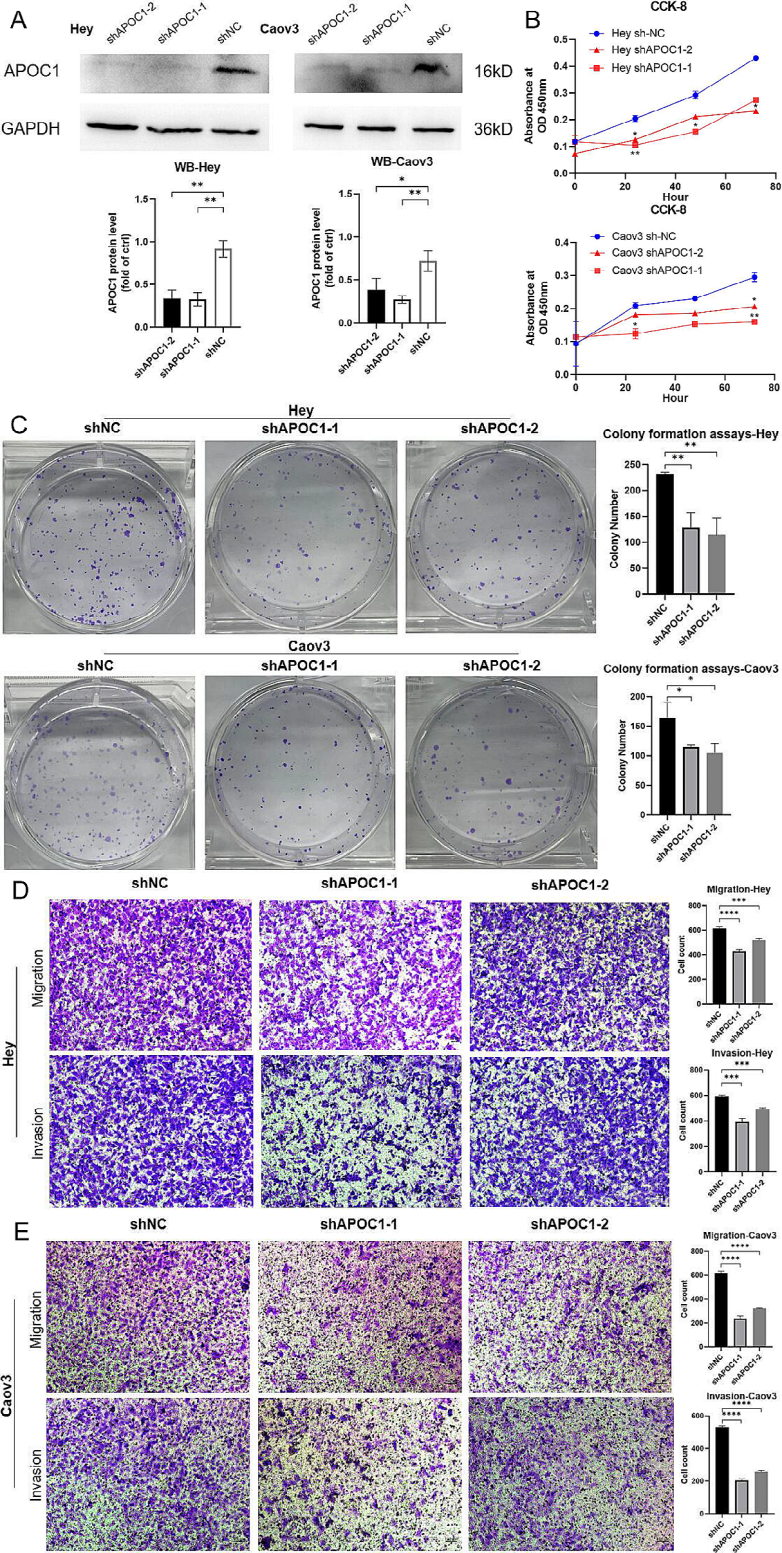
APOC1 enhances the proliferation, migration and invasion ability of HGSOC cells in vitro. (**A** ) Western blot assay of Hey and Caov3 APOC1 knockdown cells. (Uncropped images can be found in the Supplementary file) (**B** ) CCK-8 assay was performed to determine the relationship between APOC1 expression and growth ability. (**C** ) Colony formation assay was performed to determine the relationship between APOC1 expression and clone formation ability. (**D**-**E**) Migration and invasion assays were conducted to determine the relationship between APOC1 expression and cell migration and invasion ability. *: *p* < 0.05. **: *p* < 0.01 ***: *p* < 0.001. ****: *p* < 0.0001

First, a Western Blot experiment was carried out to validate the effective knockdown of APOC1 in Hey and Caov3 cells (Fig. 2A). Subsequently, a CCK-8 assay was conducted to evaluate cell proliferation, demonstrating that decreased APOC1 expression hindered cell proliferation (Fig. 2B). Furthermore, a colony formation assay indicated that reduced APOC1 expression impaired the cells’ ability to form colonies (Fig. 2C). Additionally, the cell migration assay revealed a significant inhibition of cell migration with reduced APOC1 expression. Moreover, the cell invasion assay demonstrated that decreased APOC1 expression suppressed cell invasion (Fig. 2D-E). In conclusion, these findings indicate that APOC1 plays a critical function in regulating the proliferative, migratory, and invasive capacities of OV cells in vitro.

### Potential mechanism of APOC1 in OV

**Fig. 3:**
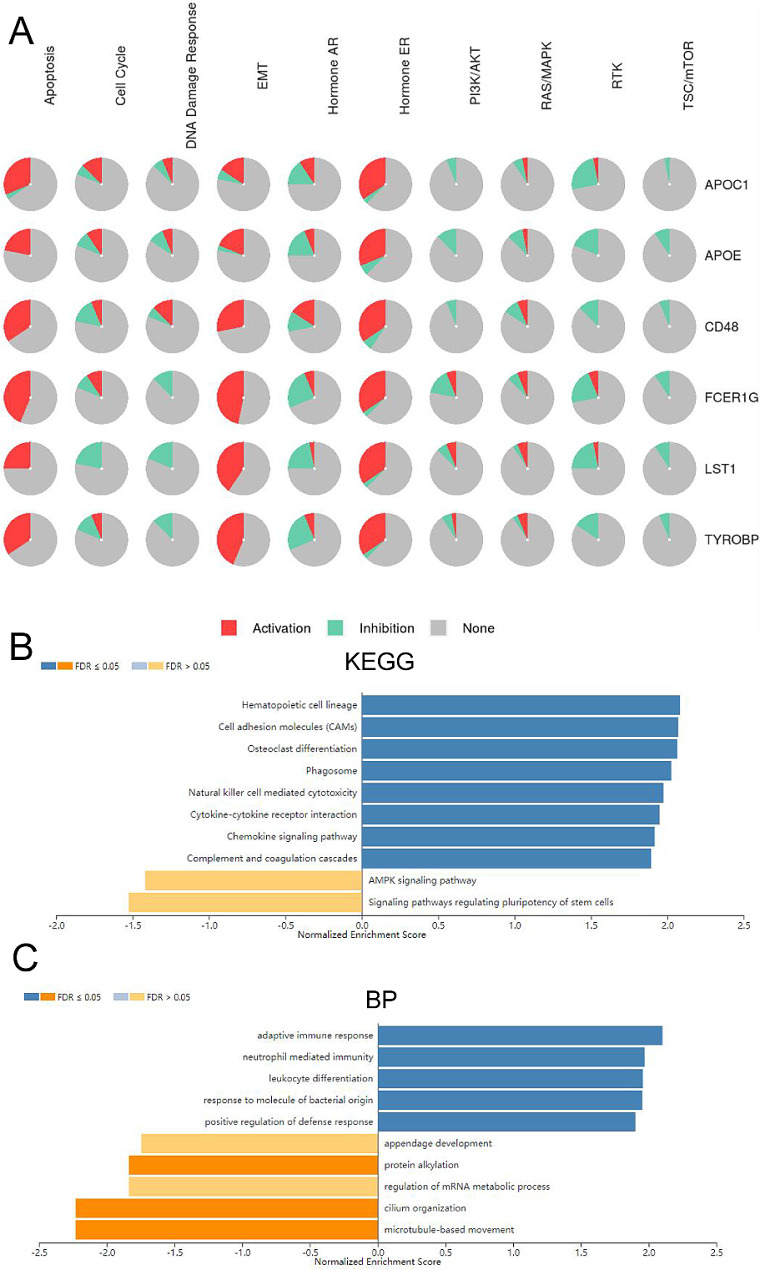
Potential mechanism of APOC1 in OV. (**A** ) The role of APOC1 in the cancer related pathways. The figure was downloaded from the GSCALite website (www.bioinfo.life.hust.edu.cn/web/GSCALite/). (**B** ) KEGG analysis of APOC1 in TCGA cohort of OV. (**C** ) Biological process analysis of APOC1 in TCGA cohort of OV. The figure B and C were downloaded from the LinkedOmics website (www.linkedomics.org)

We utilized STRING and GeneMANIA to conduct PPI network analysis of APOC1, aiming to explore potential interactions between proteins associated with APOC1. The PPI network yielded 11 nodes and 55 edges (Figure S2A). The identified APOC1-related genes were functionally linked to several processes, including cholesterol metabolism, vitamin digestion and absorption, fat digestion and absorption, and lipid particle composition. Additionally, GeneMANIA analysis revealed that the functions of these APOC1-related genes were primarily related to the remodeling of protein-lipid complexes, plasma lipoprotein particle remodeling, remodeling of protein-containing complexes, organization of plasma lipoprotein particles, organization of protein-lipid complex subunits, and lipoprotein particles (Figure S2B). To further investigate the potential mechanism of APOC1 in OV, we employed LinkedOmics and GSCALite online platforms to discover co-expressed genes and associated pathways in the TCGA data. The LinkedOmics platform results revealed that APOC1 positively correlated with 8,299 genes (depicted as red dots) and negatively correlated with 11,732 genes (depicted as green dots) in OV (Figure S2C). We have provided the top 10 genes that positively and negatively associate with APOC1 in OV in Tables 2 and 3 respectively. Notably, TYROBP (cor = 0.849397, *p* = 1.53e-85), FCER1G (cor = 0.838395, *p* = 2.56e-81), CD48 (cor = 0.812535, *p* = 1.58e-72), LST1 (cor = 0.812054, *p* = 1.00e-85), and APOE (cor = 0.808613, *p* = 2.58e-71) displayed the strongest correlation with APOC1 in OV (Figure S2D). We explored them utilizing the pathway activity module of the GSCALite platform to determine whether these six genes (TYROBP, FCER1G, CD48, LST1, APOE, and APOC1) act through specific cancer pathways. The results indicated that APOC1 could promote processes such as apoptosis, cell cycle, estrogen receptor (ER), and signaling epithelial-mesenchymal transition (EMT), while it inhibited androgen receptor (AR), PI3K/AKT, receptor tyrosine kinase (RTK), and TSC/mTOR signaling pathways (Fig. 3A). Moreover, we conducted enrichment analysis on the top 500 genes most relevant to APOC1, according to KEGG pathway analysis. The upregulated pathways included, natural killer cell-mediated cytotoxicity, osteoclast differentiation, cell adhesion molecules (CAMs), phagosome, cytokine-cytokine receptor interaction, chemokine signaling pathway, and complement and coagulation cascades. Conversely, the downregulated pathways included the AMP-activated protein kinase (AMPK) signaling pathway, signaling pathways regulating pluripotency of stem cells, and RNA transport (Fig. 3B). Additionally, GO terms revealed that the co-expressed genes of APOC1 primarily activated activities related to adaptive immune response, neutrophil-mediated immunity, response to molecule of bacterial origin, leukocyte differentiation, and positive regulation of defense response. Activities such as appendage development, protein alkylation, regulation of mRNA metabolic process, cilium organization, and microtubule-based movement were inhibited (Fig. 3C).

**Table 2:** The top-10 genes positively correlated with APOC1 in OV

Gene	Spearman Correlation	P-value	FDR(BH)
TYROBP	0.849397	1.53E-85	1.80E-83
FCER1G	0.838395	2.56E-81	3.00E-79
CD48	0.812535	1.58E-72	1.84E-70
LST1	0.812054	1.00E-85	1.00E-83
APOE	0.808613	2.58E-71	2.99E-69
GMFG	0.799471	1.37E-68	1.57E-66
HAVCR2	0.797838	4.05E-68	4.64E-66
AIF1	0.797538	1.00E-85	1.00E-83
LILRB4	0.78731	3.52E-65	4.01E-63
ABI3	0.785221	1.29E-64	1.46E-62

FDR (BH)– FDR is calculated by BH (Benjamini-Hochberg method)

**Table 3:** The top-10 genes negatively correlated with APOC1 in OV

Gene	Spearman Correlation	P-value	FDR(BH)
ZNF605	-0.52041	1.99E-22	8.29E-21
WDR35	-0.51659	4.53E-22	1.85E-20
BSN	-0.51184	1.00E-85	1.00E-83
ZNF84	-0.50279	8.11E-21	3.14E-19
ZNF638	-0.49636	2.98E-20	1.13E-18
STK36	-0.4929	5.93E-20	2.23E-18
WDR6	-0.4846	3.00E-19	1.10E-17
CHD4	-0.48243	4.56E-19	1.66E-17
HYDIN	-0.47738	1.00E-85	1.00E-83

FDR (BH)– FDR is calculated by BH (Benjamini-Hochberg method)

### Association of APOC1 expression with immune cells in OV

**Fig. 4:**
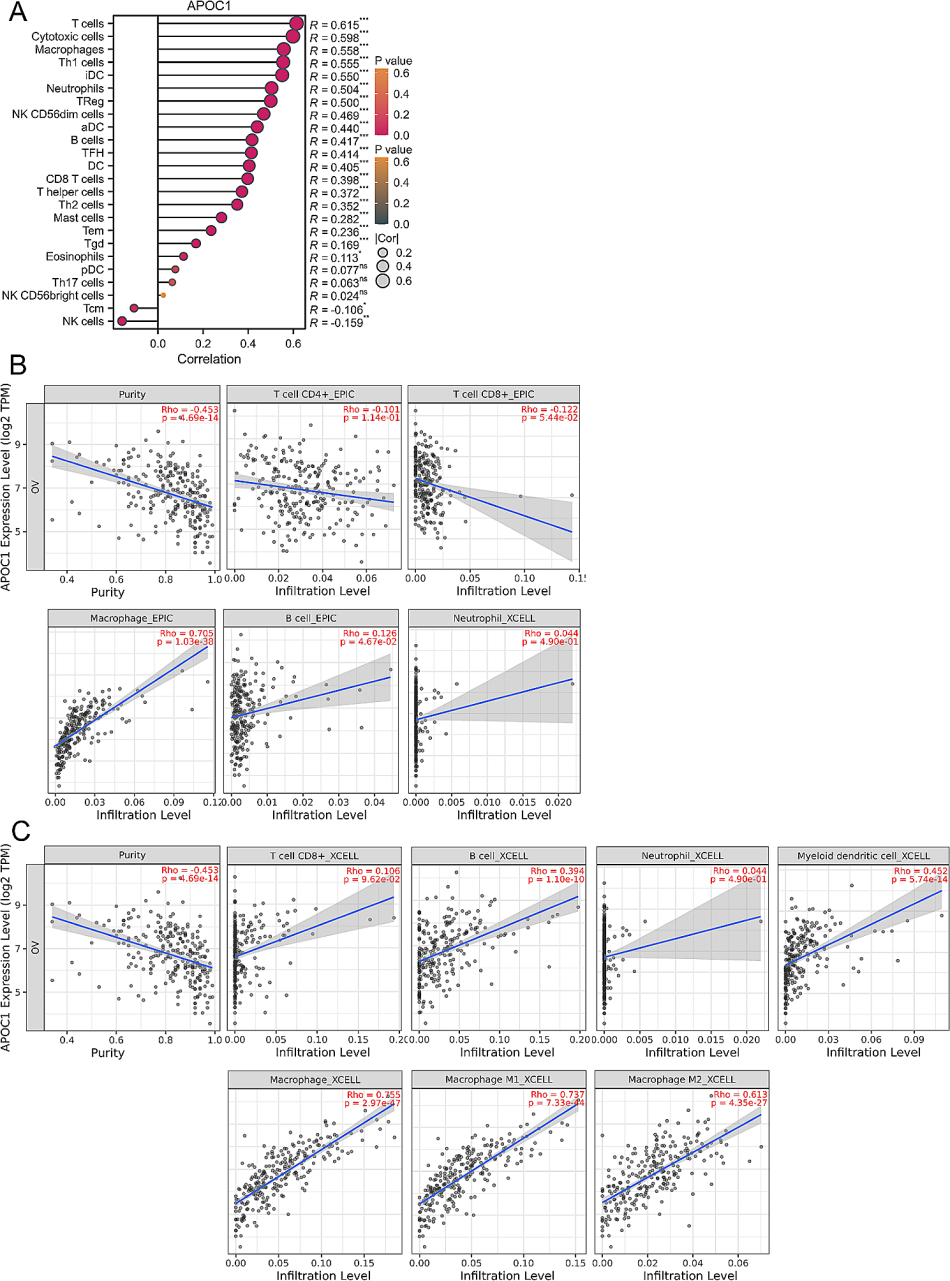
Association of APOC1 expression with immune cells in OV. (**A** ) APOC1 was most closely related to infiltration of T cells, Cytotoxic cells and Macrophages among the immune infiltration cells of OV. (**B** ) APOC1 expression was positively correlated with Macrophage (Rho = 0.705, *p* = 1.03e-38). (**C** ) The APOC1 expression was positively correlated with B cells (Rho = 0.394, *p* = 1.10e-10), Myeloid dendritic cells (Rho = 0.452, *p* = 5.74e-14), Macrophage (Rho = 0.755, *p* = 2.97e-47), Macrophage M1 (Rho = 0.737, *p* = 7.33e-44) and Macrophage M2 (Rho = 0.613, *p* = 4.35e-27). The figure was downloaded from the online platform TIMER 2.0 (http://timer.cistrome.org/)

The analysis conducted on our LinkedOmics platform suggests that the function of APOC1 in OV primarily revolves around the adaptive immune response. Therefore, it is possible that APOC1 is linked to the immune response in OV. Through immune infiltration analysis of TCGA data (Fig. 4A), we observed that T cells, cytotoxic cells, and macrophages exhibited the highest correlation with APOC1 expression. To explore further the association between the expression of APOC1 and immune cells, we utilized the TIMER 2.0 platform. Using the EPIC algorithm, we discovered a strong positive correlation between APOC1 expression and macrophages (Rho = 0.705, *p* = 1.03e-38) (Fig. 4B). Moreover, employing the XCELL algorithm, we determined that APOC1 expression positively correlated with B cells (Rho = 0.394, *p* = 1.10e-10), myeloid dendritic cells (Rho = 0.452, *p* = 5.74e-14), macrophages (Rho = 0.755, *p* = 2.97e-47), M1 macrophages (Rho = 0.737, *p* = 7.33e-44), and M2 macrophages (Rho = 0.613, *p* = 4.35e-27) (Fig. 4C). These findings suggest a specific association between APOC1 expression and macrophage cells.

### APOC1 is associated with M2 phenotypic TAMs in OV

**Fig. 5:**
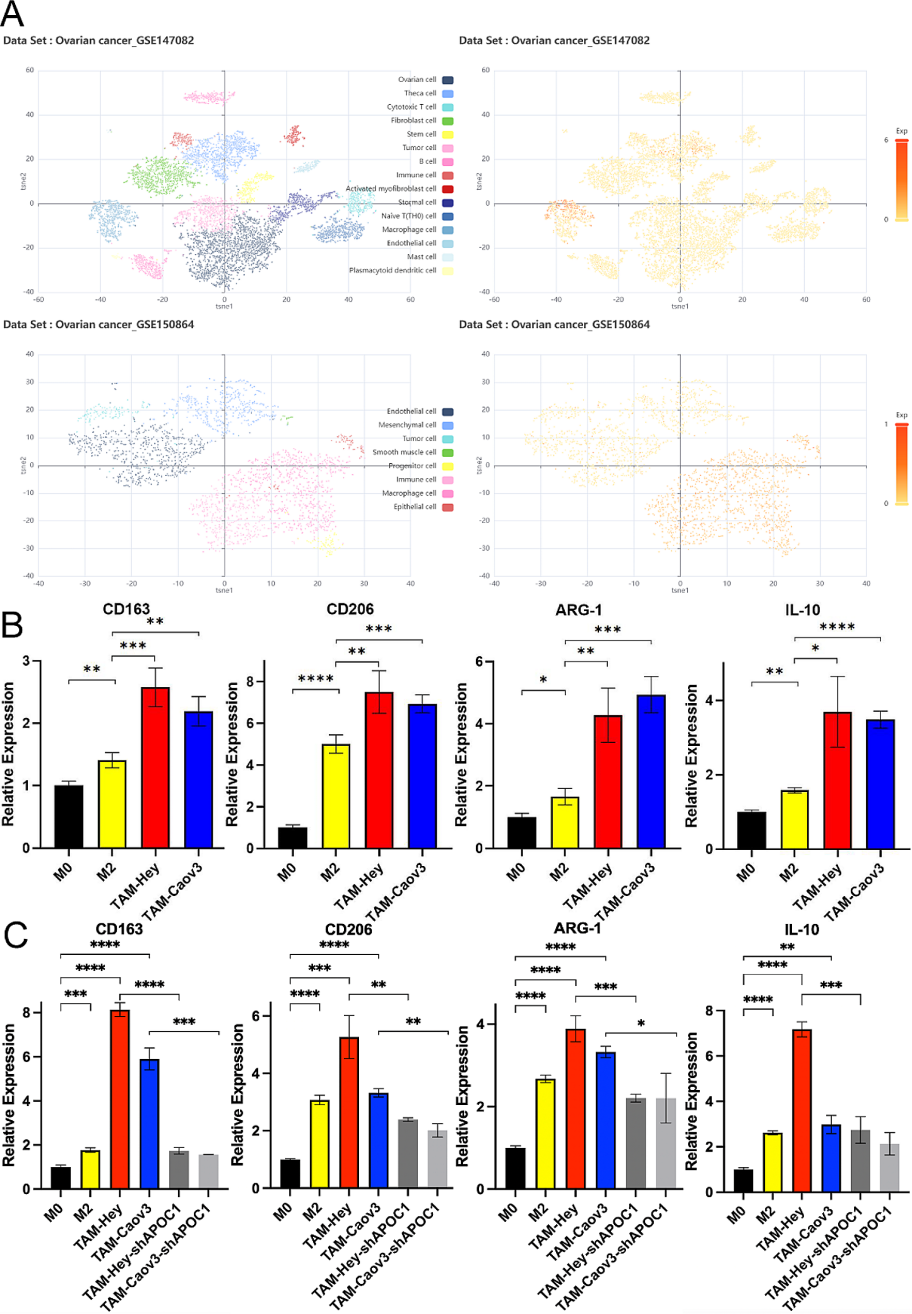
APOC1 is associated with M2 TAMs in OV. (**A**) Single-cell sequencing data GSE147082 and GSE150864 indicate that APOC1 is predominantly enriched in macrophages in OV. (**B**) Elevated expression of M2 markers CD163, CD206, IL10 and ARG1 in TAMs associated with ovarian cancer cells. (**C**) Reduced expression of M2 markers CD163, CD206, IL10 and ARG1 in TAMs associated with APOC1 knockdown cells in OV. *: *p* < 0.05. **: *p* < 0.01 ***: *p* < 0.001. ****: *p* < 0.0001

Based on these findings, we conducted further investigations into the relationship between APOC1 and macrophages in OV. Through the analysis of single-cell sequencing data from GSE147082 and GSE150864 (Fig. 5A), we discovered that APOC1 was predominantly enriched in macrophages in OV. To gain a deeper understanding, we established an in vitro model of TAMs. Initially, THP-1 cells were treated with PMA to convert them into M0 macrophages. Subsequently, a portion of these cells were further treated with IL-13 and IL-4 for 48 h to induce the conversion into M2 macrophages. Another portion of the M0 macrophages were co-cultured with ovarian cancer cells (Hey and Caov3 cells) for 48 h, leading to their transformation into TAMs. Using a qPCR assay, we assessed the expression levels of common markers of M2 macrophages, namely CD163, CD206, IL10, and ARG1. The results indicated a high expression of CD163, CD206, IL10, and ARG1 in TAMs (Fig. 5B). Additionally, we employed an APOC1 knockdown cell line derived from OV to transform TAMs. Notably, the qPCR results showed a decrease in the expression of CD163, CD206, IL10, and ARG1 in these TAMs (Fig. 5C). Collectively, these findings suggest an association between APOC1 and M2 TAMs in OV.

**Fig. 6:**
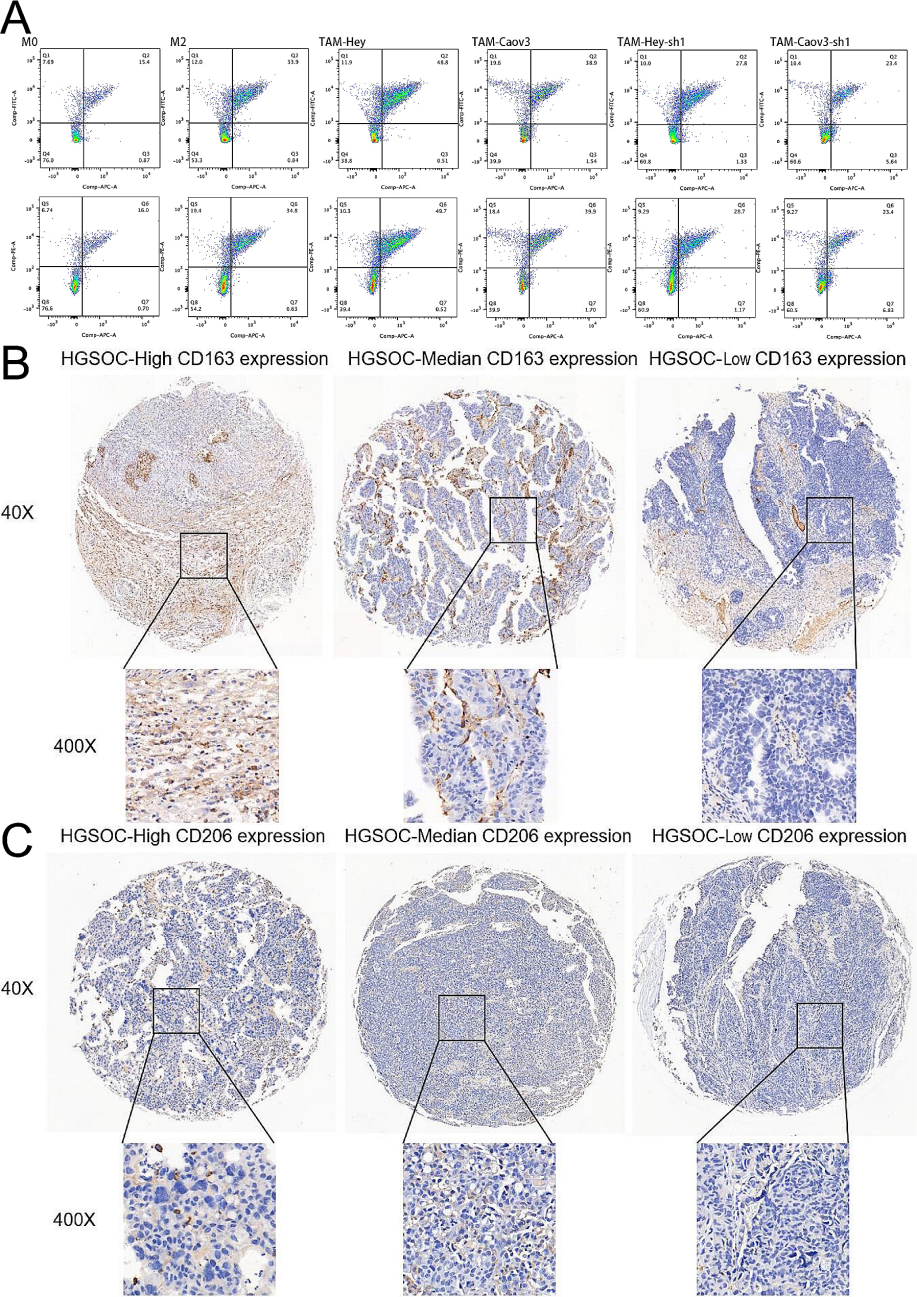
APOC1 is associated with M2 macrophage markers in OV. (**A**) Flow cytometric analysis of M2 macrophage markers CD206, CD163 and ARG1 in TAMs associated with ovarian cancer cells. (APC: CD206, FITC: CD163, PE: ARG1) (**B**) Representative figures of IHC staining of CD163 expression levels in tissue microarray (×40: scale bar = 100 μm; ×400: scale bar = 10 μm). (**C**) Representative figures of IHC staining of CD206 expression levels in tissue microarray (×40: scale bar = 100 μm; ×400: scale bar = 10 μm)

Subsequently, flow cytometry assays were performed, and the results validated the conclusions of our previous assays (Fig. 6A). Following this, IHC staining of tissue microarrays for CD163 and CD206 was conducted, and the AOD values of APOC1, CD163, and CD206 were calculated (Fig. 6B-C, S1B-C). A correlation analysis was then conducted, revealing a significant correlation between the expression of APOC1 and CD163 (*p* = 0.0083), while no significant correlation was observed with the expression of CD206 (*p* = 0.4184) (Table 4).

**Table 4:** Spearman association analysis between APOC1 and CD163 and CD206

Variables	APOC1 expression level	
	Spearman association	P-value
CD163 expression level	0.3353	0.0083
CD206 expression level	0.1055	0.4184

## Discussion

Tumor promotion inflammation represents a significant characteristic of tumors, whereby the inflammatory response provides the tumor microenvironment with various bioactivating molecules. These molecules encompass growth factors that sustain cancer cell proliferation and angiogenic factors that facilitate vascular growth, tumor metastasis, and invasion [38]. Among these factors, TAMs are a type of macrophages that infiltrate tumor tissues or accumulate in the microenvironment of solid tumors. TAMs possess the ability to influence tumor growth, angiogenesis, immunomodulation, and drug resistance [39]. While several current articles have established the association between APOC1 and tumor immunomodulation, as well as the phenotypic transition of macrophages, there is a lack of exploration regarding the function of APOC1 in OV. In this study, combining biological information analysis with in vitro cell assays, we aim to investigate the potential association between APOC1 and tumor immunity, as well as cancer promotion.

To begin with, we conducted an analysis of somatic mutations and CNV of APOC1 across multiple cancer types. Among these, ovarian cancer ranked sixth in terms of APOC1 alterations. Furthermore, by analyzing data from GEO, including GSE12470, GSE52037, GSE66957, and GSE10971, we observed higher expression levels of APOC1 in OV compared to normal tissues. To validate this finding, we performed immunohistochemical staining on a tissue microarray, which confirmed the elevated expression of APOC1. Previous studies have suggested that APOC1 may serve as a potential biomarker for various cancers [17, 40–42], and our analysis indicates its potential as a biomarker for OV as well. Additionally, there have been reports indicating that APOC1 promotes the progression of gastric cancer and the proliferation of prostate cancer [15, 43]. In our in vitro cellular assays, we discovered that APOC1 promotes the proliferation, migration, and invasive abilities of ovarian cancer cells.

Following that, we performed an analysis of functional enrichment on the top 500 APOC1-related genes. This analysis revealed that these APOC1-related genes are primarily involved in adaptive immune response, neutrophil mediated immunity, leukocyte differentiation, and the response to molecules of bacterial origin. These findings led us to suspect that APOC1 may be associated with the immune response in OV. In our analysis of the association between APOC1 and immunocytes, we discovered a strong association between APOC1 and macrophages in OV. Additionally, using single-cell sequencing data (GSE147082 and GSE150864), we found that APOC1 was predominantly enriched in macrophages within the OV microenvironment. Subsequently, we constructed an in vitro model of TAMs in OV and analyzed the expression of common markers in M2 macrophages through qPCR and flow cytometry. Remarkably, we observed a correlation between APOC1 and markers such as CD163, CD206, IL10, and ARG1 in M2 macrophages. Based on these findings, we hypothesized that APOC1 may be associated with M2 macrophages in OV. We subsequently conducted IHC staining of tissue microarrays for CD163 and CD206, followed by a correlation analysis with APOC1. The analysis revealed a correlation between the expression of APOC1 and CD163, but not with the expression of CD206.

In studies on inflammation, M1 macrophages are typically believed to promote inflammation, whereas M2 macrophages are generally believed to inhibit inflammation. However, this is different in TAMs. Due to the crucial role of immune escape in tumors, M2 macrophages could suppress inflammation and enhance tumor proliferation. Consequently, M2 macrophages have garnered significant attention in tumor research. Activation of M2 macrophages can give rise to four distinct types: M2a, M2b, M2c, and M2d. M2a macrophages enhance endocytosis, M2b macrophages produce pro-inflammatory and anti-inflammatory cytokines, M2c macrophages phagocytose apoptotic cells, and M2d macrophages promote angiogenesis and tumor progression [44, 45]. This highlights the significance of M2 macrophage polarization in tumors. It remains to be investigated in future studies whether there is an association between APOC1 and the polarization of M2 macrophages in OV. However, our study demonstrated an association between APOC1 and M2 macrophages in OV. APCO1 holds potential as a prognostic biomarker.

### Electronic supplementary material

Below is the link to the electronic supplementary material.

Supplementary Material 1

Supplementary Material 2

## Data Availability

The FPKM files of RNA-seq transcriptome data used to support the findings of this study were obtained from the TCGA database. The Matrix files in SOFT format for four GEO datasets (GSE12470, GSE66957, GSE10971 and GSE52037) used to support the findings of this study were obtained from in the Gene Expression Omnibus. The two single-cell sequencing datasets (GSE147082 and GSE165404) used to support the results of this study were obtained from the Gene Expression Omnibus. The data of somatic mutation and CNV of APOC1 in OV were carried out from the TCGA database.

## Abbreviations

HGSOC: High grade serous ovarian cancer
OV: Ovarian Cancer
TAMs: Tumor-associated Macrophages
APOC: Apolipoprotein C
VLDL: low-density lipoprotein
HDL: high-density
LDL: low-density lipoprotein
APOC1: Apolipoprotein C1
PPI: Protein and protein interaction
shRNA: short hairpin ribonucleic acid
FPKM: Fragments per kilobase of exon model per million mapped fragments
TCGA: The Cancer Genome Atlas
GEO: Gene Expression Omnibus
CNV: Copy number variation
GO: Gene Ontology
KEGG: Kyoto Encyclopedia of Genes and Genomes
GSEA: Gene set enrichment analysis
RNA-seq: RNA sequencing
FT: Fallopian tubes
FTE: Fallopian tube epithelium
IOD: Integrated Optical Density
AOD: Average Optical Density
