# Supplementary material for: APOC1 is a prognostic biomarker associated with M2 macrophages in ovarian cancer
Source: BMC Cancer. 2024 Mar 21;24:364. doi: 10.1186/s12885-024-12105-z (PMC10956310; doi:10.1186/s12885-024-12105-z)
Supplement: Supplementary file 1 — Supplementary Material 1 [file 12885_2024_12105_MOESM1_ESM.pdf]

Supplementary figures and table

Table S1 Primers' sequences used in qPCR were as follows:

Gene Symbol	Primers' sequences
GAPDH	Forward primer: 5'-CAGGGCTGCTTTTAACTCTGGTAA-3'
	Reverse primer: 5'-GGGTGGAATCATATTGGAACATGT-3'
CD163	Forward primer: 5'-TTTGTCAACTTGAGTCCCTTCAC-3'
	Reverse primer: 5'-TCCCGCTACACTTGTTTTCAC-3'
CD206	Forward primer: 5'- TCCGGGTGCTGTTCTCCTA-3'
	Reverse primer: 5'-CCAGTCTGTTTTTGATGGCACT-3'
IL-10	Forward primer: 5'- GACTTTAAGGGTTACCTGGGTTG-3'
	Reverse primer: 5'-TCACATGCGCCTTGATGTCTG-3'
ARG1	Forward primer: 5'-GTGGAAACTTGCATGGACAAC-3'
	Reverse primer: 5'-AATCCTGGCACATCGGGAATC-3'

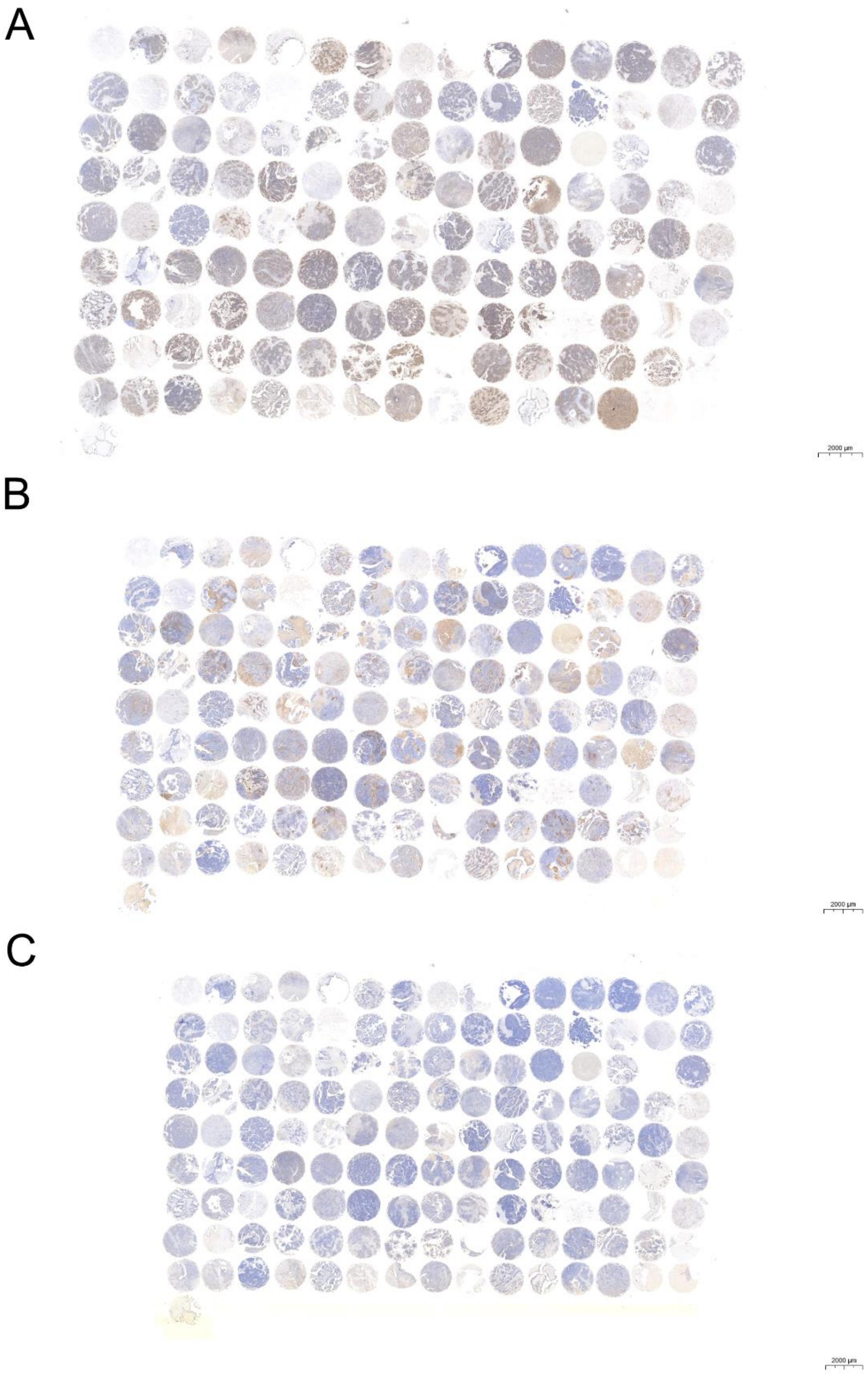

Figure S1 (A) APOC1 immunohistochemistry analysis of tissue microarray consisting of 27 normal ovarian (13 FT tissues) and 93 ovarian cancer (61 HGSOC tissues) paraffin sections. (B) CD163 immunohistochemistry analysis of tissue microarray consisting of 27 normal ovarian (13 FT tissues) and 93 ovarian cancer (61 HGSOC tissues) paraffin sections. (C) CD206 immunohistochemistry analysis of tissue microarray consisting of 27 normal ovarian (13 FT tissues) and 93 ovarian cancer (61 HGSOC tissues) paraffin sections.

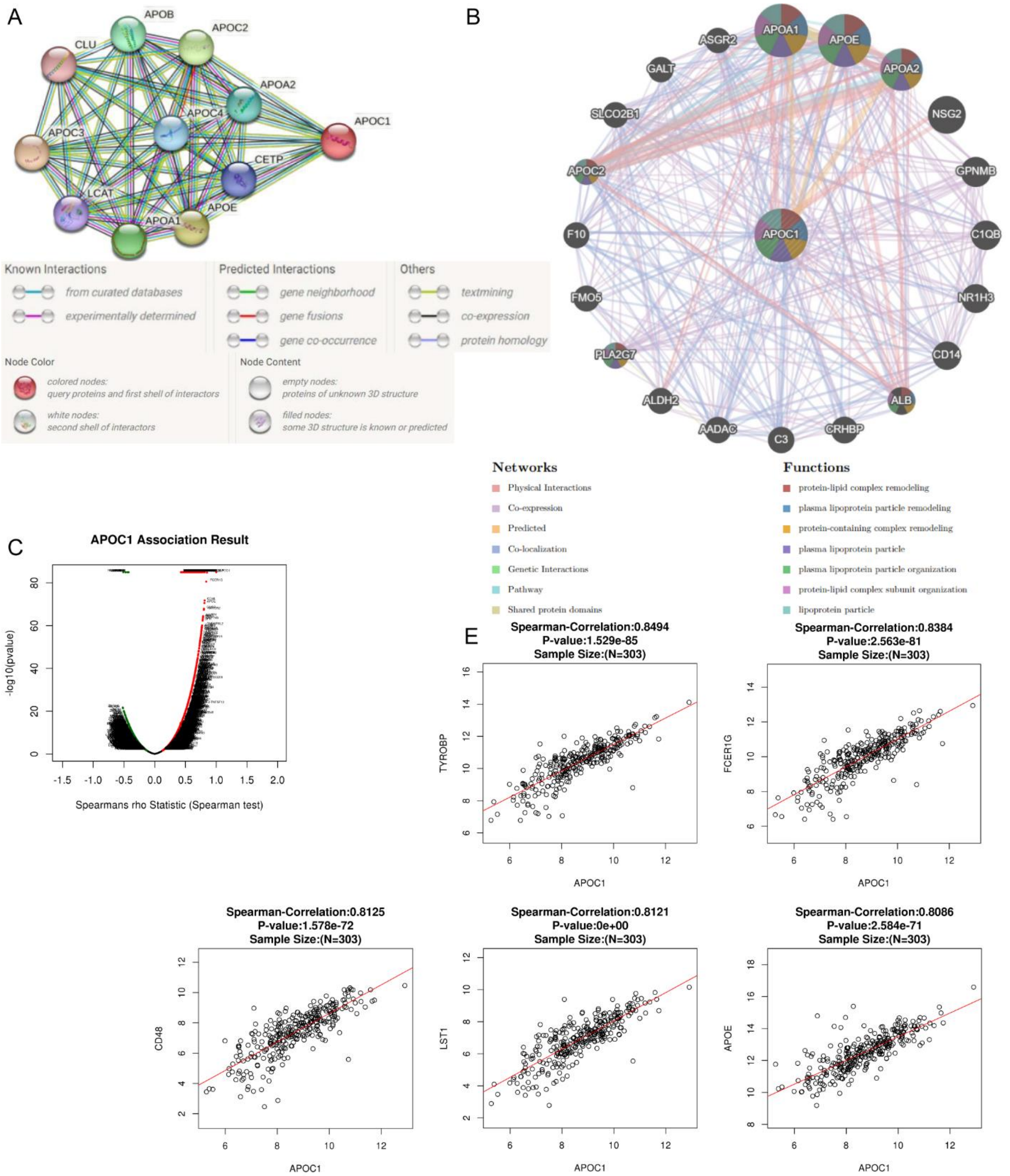

Figure S2 PPI interaction network and co-expression analysis of APOC1 in OV.

(A) Protein-protein interaction network of APOC1 of STRING. The figure was downloaded from the online tool STRING 11.0 b (https://string-db.org/). (B) Protein-protein interaction network of APOC1 of GeneMANIA. The figure was downloaded from the online tool GeneMANIA (http://www.genemania.org). (C) The genes positively and negatively correlated with APOC1 in OV. (D) Correlation of TYROBP, FCER1G, CD48, LST1 and APOE with APOC1. The figure C and D were downloaded from the LinkedOmics website (www.linkedomics.org).
