# Supplementary material for: APOC1 is a prognostic biomarker associated with M2 macrophages in ovarian cancer
Source: BMC Cancer. 2024 Mar 21;24:364. doi: 10.1186/s12885-024-12105-z (PMC10956310; doi:10.1186/s12885-024-12105-z)
Supplement: Supplementary file 2 — Supplementary Material 2 [file 12885_2024_12105_MOESM2_ESM.pdf]

Supplementary original figures of Western blotting

After specific detection of the target antigen confirmation, we cut between 25kDa - 35kDa and incubated the upper half with anti-GAPDH polyclonal antibody and the lower half with anti-APOC1 polyclonal antibody. In the article show images we marked with red boxes.

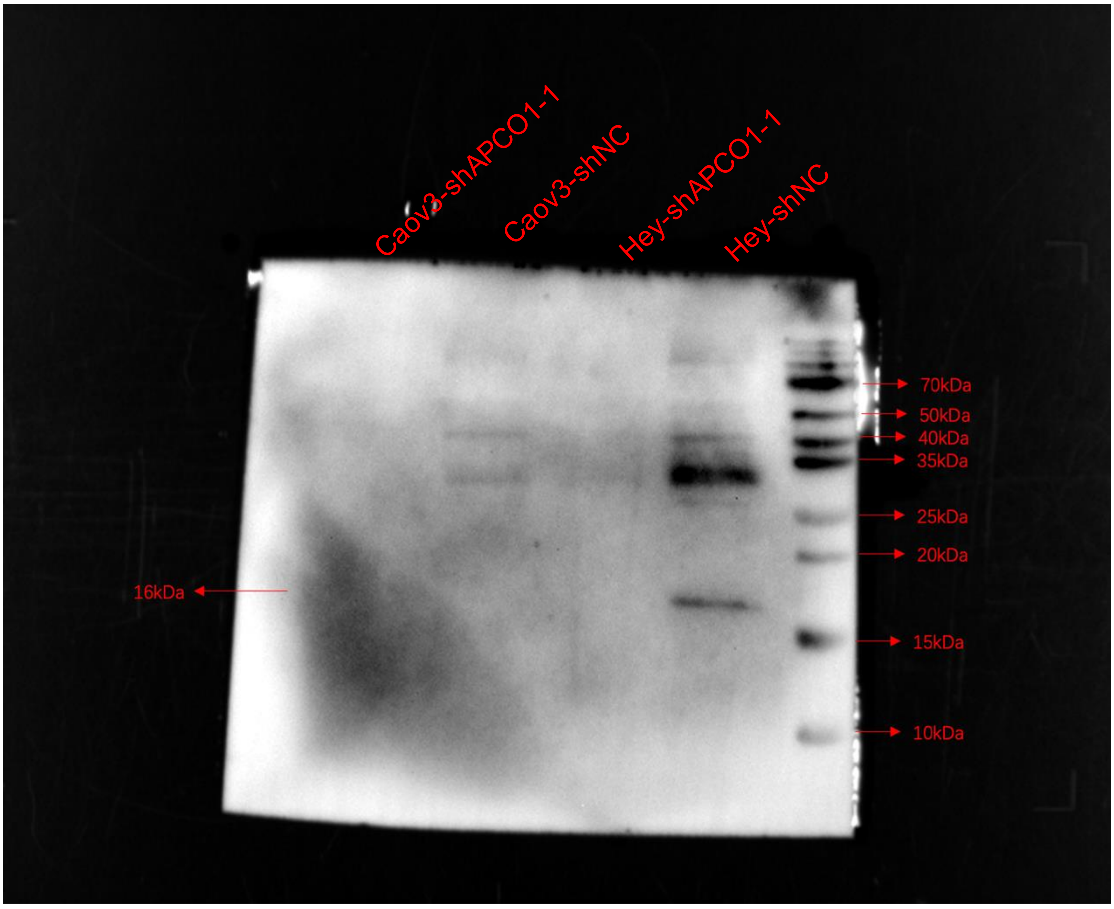

Figure 1 Fuller-length, original, and unprocessed Western blot images of Anti-APOC1 antibody to confirm specific detection of the target antigen. (Predicted band size: 9 kDa, Observed band size: 16 kDa)

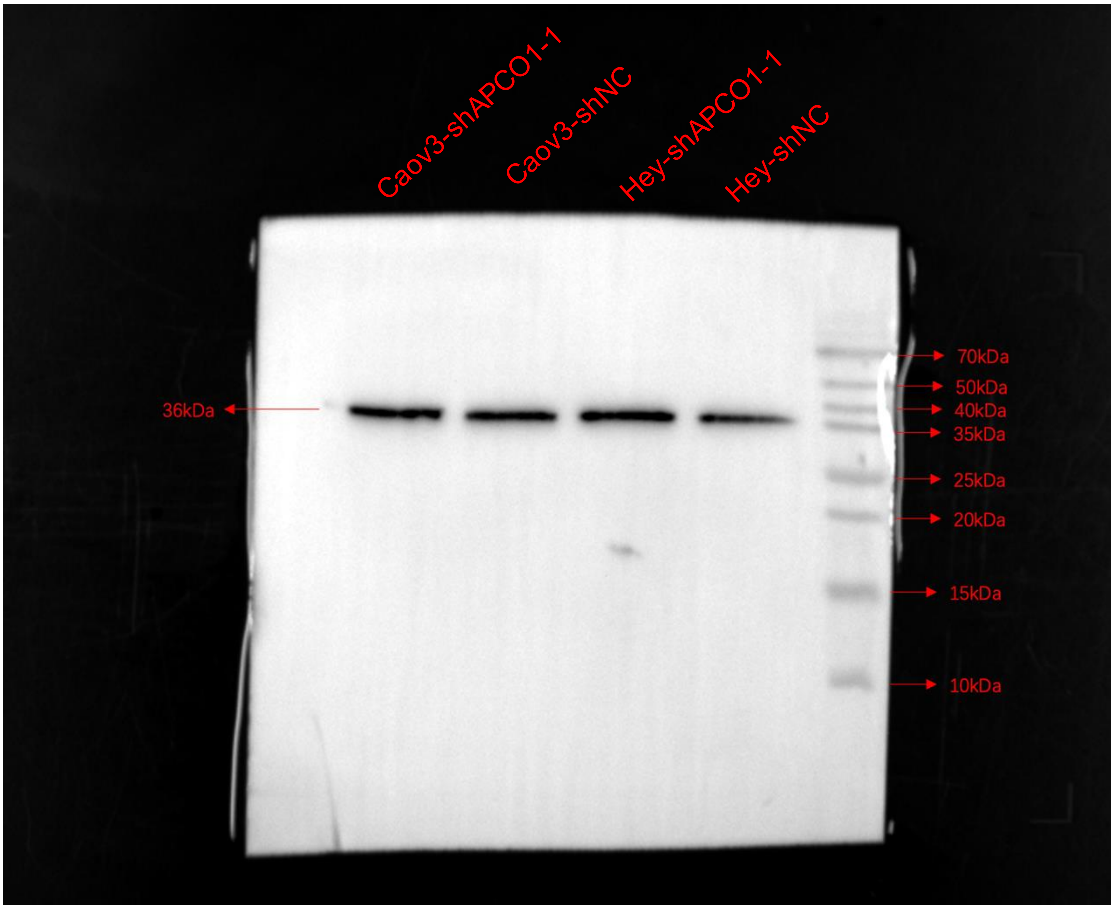

Figure 2 Fuller-length, original, and unprocessed Western blot images of Anti- GAPDH antibody to confirm specific detection of the target antigen. (Predicted band size: 36 kDa, Observed band size: 36 kDa)

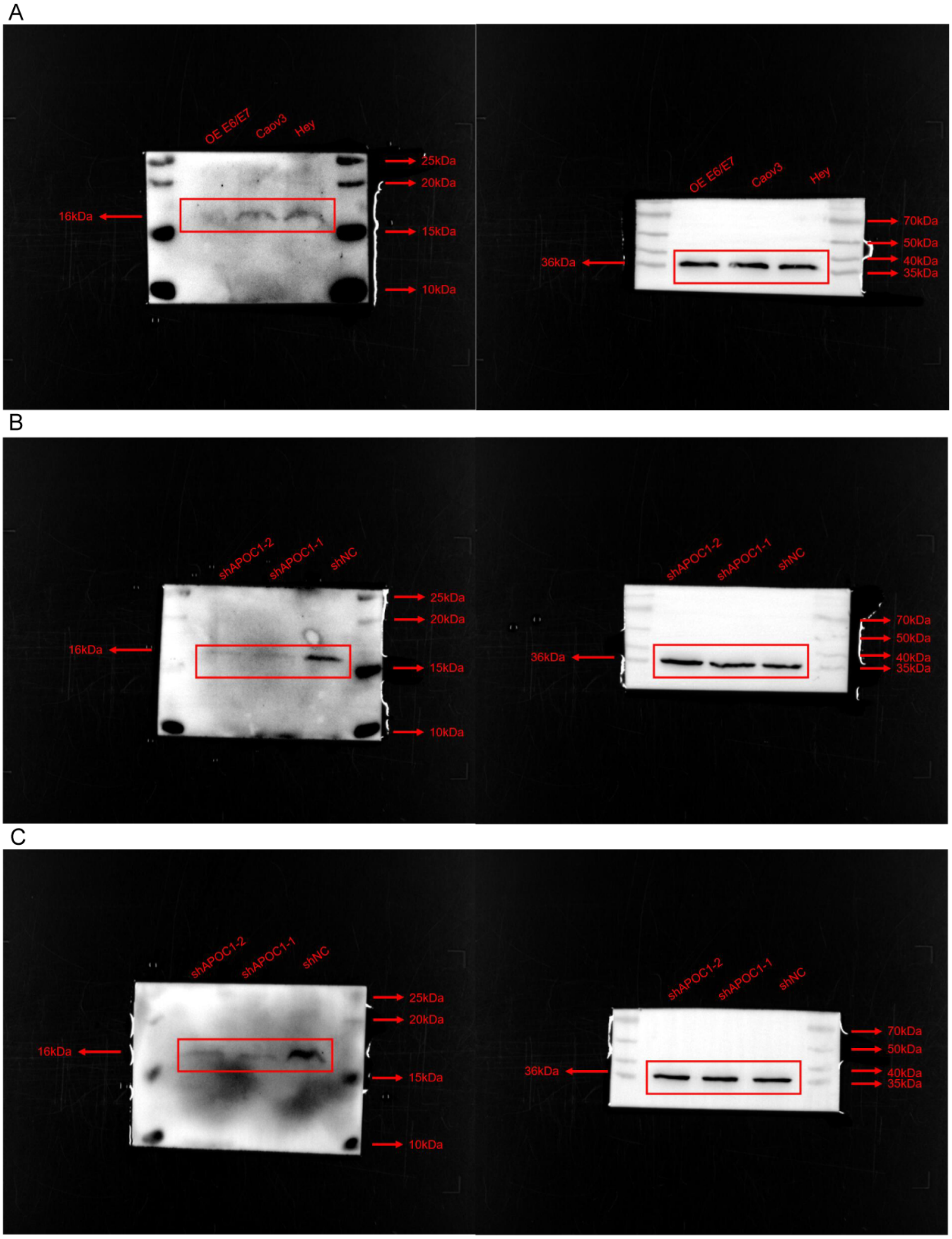

Figure 3 Western Blot assay of Figure 1C and Figure 2A .

(A) The original Western blot images of Figure 1C. (B) The original Western blot images of Hey in Figure 2A. (C) The original Western blot images of Caov3 in Figure 2A. (The region of the original blots used in Figure 1C and Figure 2A has been denoted using red box.)

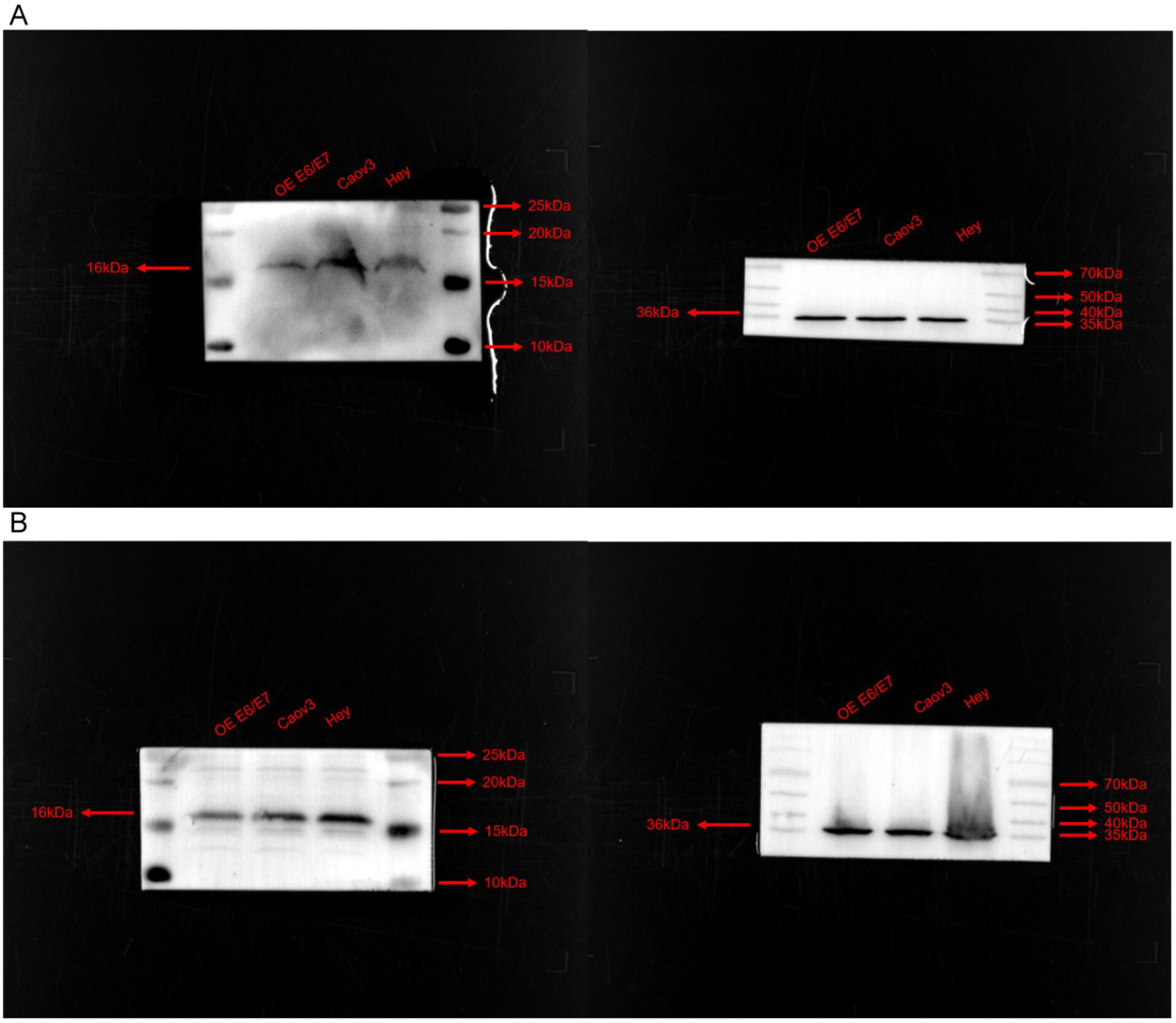

Figure 4 Replicate Western blot analysis of APOC1 in Hey, Caov3 and OE E6/E7 cells

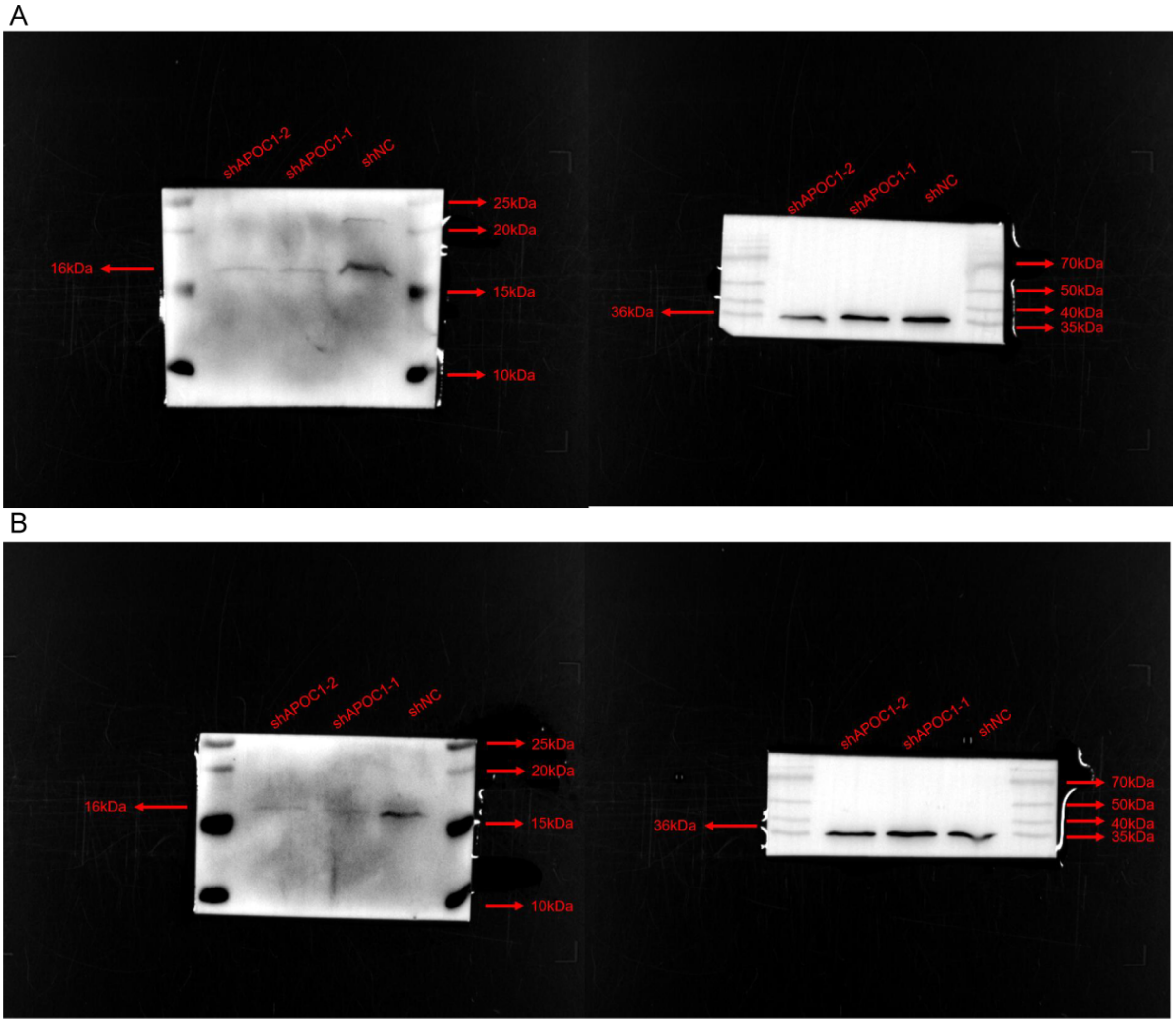

Figure 5 Replicate Western blot assay of Hey APOC1 knockdown cells.

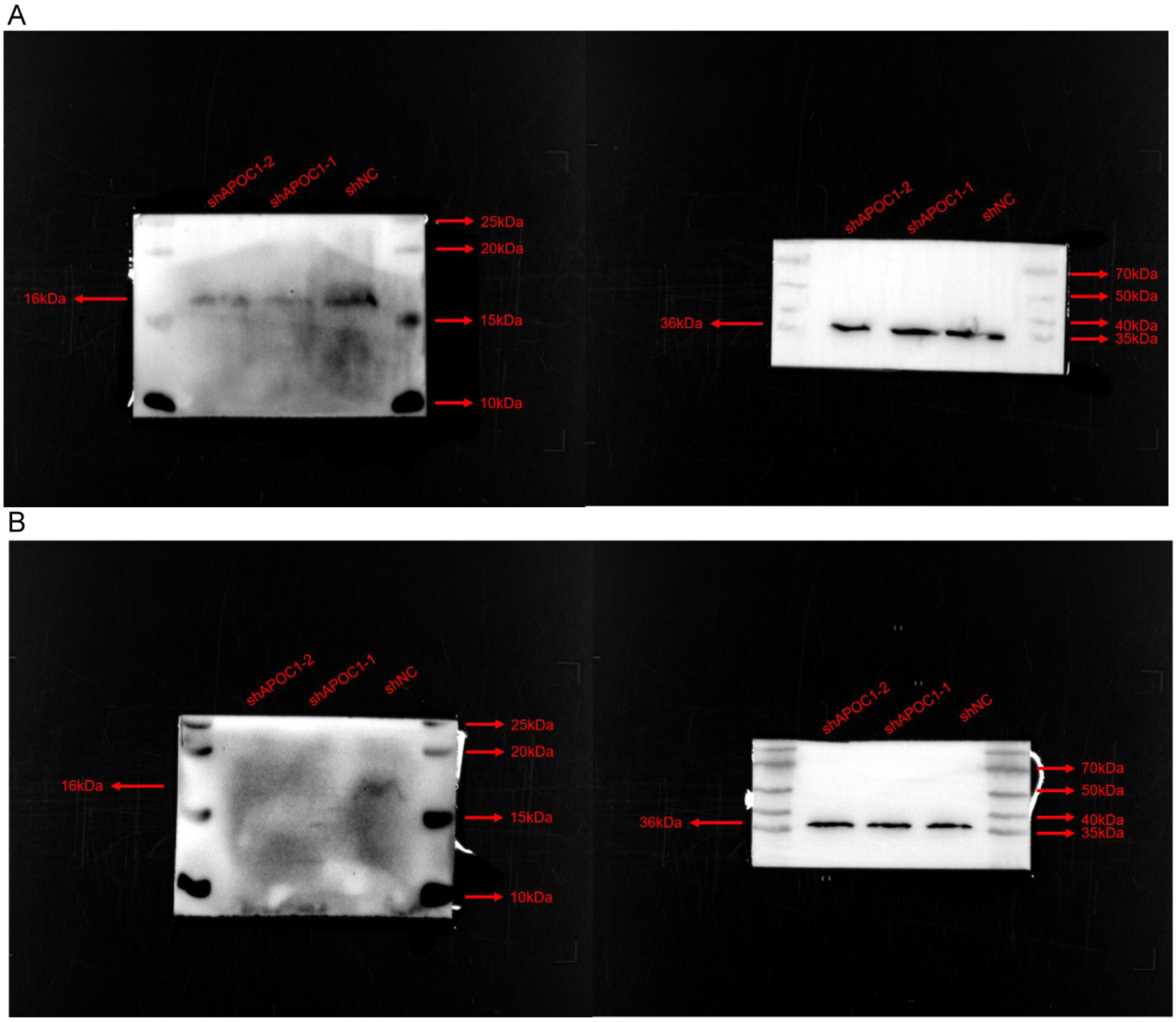

Figure 6 Replicate Western blot assay of Caov3 APOC1 knockdown cells.
